# Behaviour of Corroded Single Stud Shear Connectors

**DOI:** 10.3390/ma10030276

**Published:** 2017-03-09

**Authors:** Wen Xue, Ju Chen, Ji-Hua Zhu

**Affiliations:** 1 School of Civil Engineering and Architecture, Zhejiang University of Science and Technology, Hangzhou 310023, China; xuewen@zust.edu.cn; 2 Department of Civil Engineering, Zhejiang University, Hangzhou 310058, China; cecj@zju.edu.cn; 3 Guangdong Provincial Key Laboratory of Durability for Marine Civil Engineering, Shenzhen University, Shenzhen 518060, China

**Keywords:** corrosion, loading test, push test specimen, single stud shear connector, test setup

## Abstract

In this study, the effect of corrosion on the static behavior of stud shear connectors was investigated. An innovative test setup for single stud shear connectors was designed and established. Two series of specimens having different stud diameters were fabricated and tested. The test specimens were firstly corroded to different corrosion rates by the electronic accelerating method. Static loading tests were then performed to obtain the load-slip curves and ultimate strengths of the corroded test specimens. The actual corrosion rates were measured from the studs obtained from the tested specimens. The test results were compared with the push out test specimens having similar corrosion rates. It is shown that the test results obtained from the single stud shear connectors are conservative compared with the corroded push test specimens, which prove the validation of the single stud shear connector test method. The effect of corrosion on the behavior of stud shear connectors was also presented.

## 1. Introduction

The economic loss caused by corrosion in concrete structures is tremendous. Therefore, understanding the effect of corrosion is crucial to predicting the behavior of concrete structures in use. Many studies have been conducted to evaluate the effect of the corrosion of reinforcing bars on concrete structures [1,2,3,4,5]. However, there are few studies that have been conducted on the effect of corrosion on the behavior of stud shear connectors used in steel-concrete composite beams [6,7].

Steel-concrete composite beams are developed structures based on RC structures and steel structures, and are nowadays widely used in buildings and bridge constructions due to the satisfying utilization of the two materials. However, unfavorable conditions may cause corrosion to occur in the interface between the steel and concrete since there is a lack of protection. Headed stud shear connectors are the most common type of shear connectors and are used in composite bridges. The behavior of the stud connectors has been broadly investigated by many researchers [8,9,10,11,12,13]. The deterioration in strength of stud connectors due to fatigue damage has also been reported [14,15,16,17,18,19,20,21]. Chen [6] has investigated the behavior of corroded shear stud connectors based on push out test specimens. However, the corrosion rates of four stud shear connector push out test specimens were different. The single stud shear connector test has been used recently [22,23]. In this study, an innovative test setup for single stud shear connectors was proposed to accurately evaluate the effect of corrosion.

## 2. Experimental Investigation

### 2.1. Test Specimens

**Figure 1:**
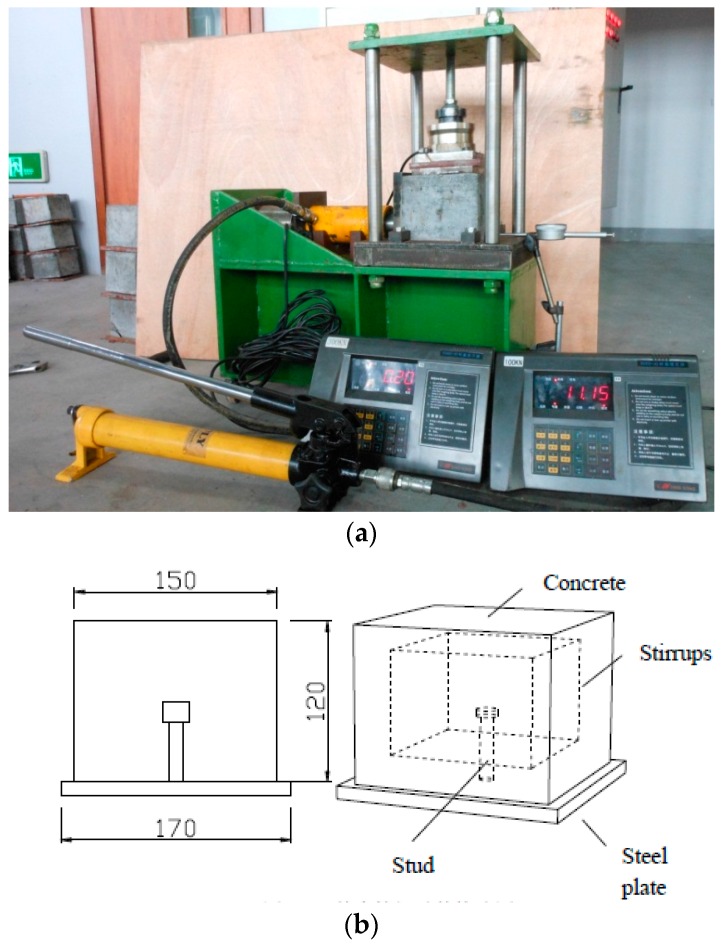
Test setup of the single stud shear connector: (**a**) Test device; (**b**) Test specimen.

The proposed test device is shown in Figure 1a. Both the horizontal force and vertical force on the test specimen was measured. The horizontal force was applied by a hand jack. Figure 1b shows the details of the test specimens. The stirrups are HPB 235 (Mengruidi Steel Company, Hangzhou, China) with a diameter of 6 mm. The test specimens were labeled so that the corrosion state, nominal stud diameter, and expected corrosion rate could be identified from the label. For example, the labels “W10.0-5” and “B16.0-10” define the specimens as follows:

- The first letter indicate that the designed corrosion state, where the prefix letter “W” refers to corrosion along the whole stud shank, while the letter “B” refer to corrosion only at the bottom of the stud shank.
- The following three digits (10.0 and 13.0) indicate the nominal diameter of the studs in mm.
- The following one (5) or two digits (10) are the expected corrosion rates of the stud in percentage.

### 2.2. Material Properties and Measurements

Three concrete cubic specimens were prepared at the time of the push test specimen casting, to determine the concrete strength of the push test specimens. Table 1 summarizes the material properties of concrete at 28 days. Two kinds of studs with nominal diameters of 10.0 and 13.0 mm were used in this study. The heights of the studs are 50 mm and 70 mm, respectively. Tensile tests for the stud material were conducted. The yield stress from the tensile tests was determined by 0.2% strain because the steel used for studs generally does not show a clear yielding point. Table 2 summarizes the material properties of the stud material. Quality control of the welding process is a very important factor, since the effect of welding quality may cover the effect of corrosion. Therefore, welding trials were carried out to obtain the proper and reliable welding quality.

### 2.3. Accelerating Corrosion Process

**Figure 2:**
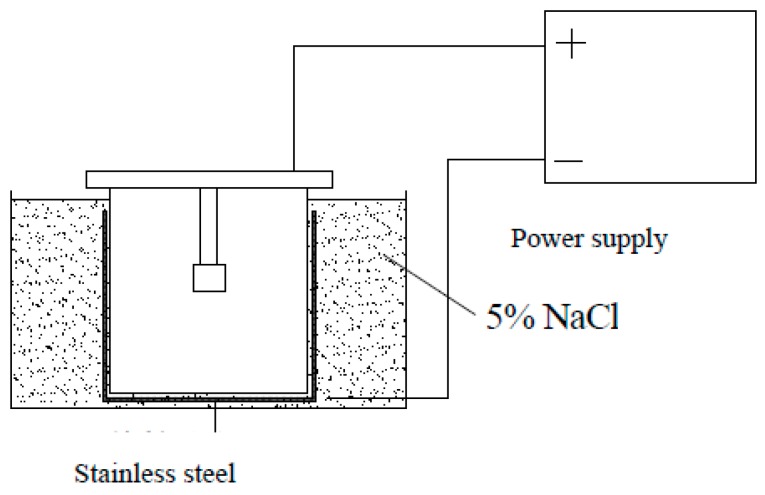
Setup of the electronic accelerating corrosion.

All specimens, except the uncorroded one (control specimen), were immersed in a 5% NaCl solution for three days after being cured for 28 days, and then the direction of current (about 0.2 μA/cm^2^) was arranged for accelerating stud corrosion; studs worked as the anodes, while a piece of stainless steel positioned in the solution served as the cathode, as shown in Figure 2. The corrosion time of each specimen was determined based on the expected corrosion rate. Faraday’s theory was used to calculate the corrosion time. The calculated results are shown in Table 3 and Table 4 for the 10.0 mm series and 13.0 mm series, respectively. The actual corrosion time was the same as the calculated result. It should be noted that the actual corrosion rates of test specimens may differ from those expected corrosion rates. 

### 2.4. Loading Test Setup and Procedure

**Figure 3:**
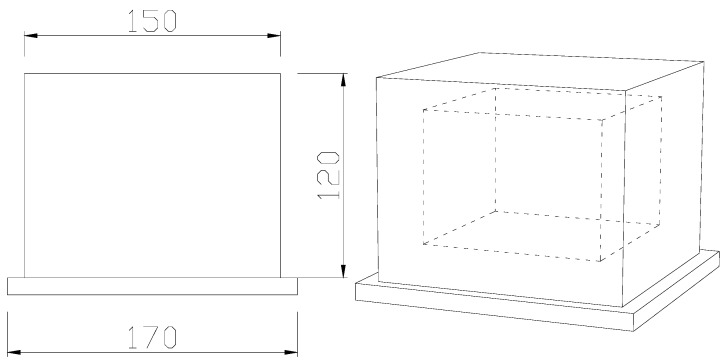
Test specimens for the friction test.

**Figure 4:**
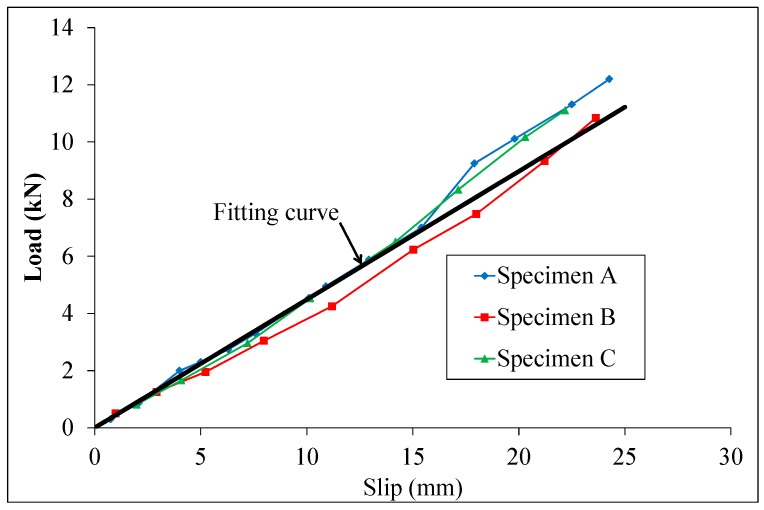
Load-slip curves of the specimens without studs.

Corroded push test specimens were loaded in the test device shown in Figure 1. The horizontal and vertical forces were measured. The measured ultimate strengths of the specimens are shown in Table 5 and Table 6. The slip between the steel member and the two slabs was measured using LVDTs. In this study, the expected failure load of the corroded specimens was difficult to predict, therefore the load was first applied in increments up to 10% of the failure load of specimens having a 5% less expected corrosion rate. Subsequent load increments were then imposed such that failure does not occur in less than 15 minutes and the approximate loading rate was 0.5 mm/min. The longitudinal slip between each concrete slab and the steel section was measured at each load increment. The friction between the concrete block and steel plate was obtained by the specimen without studs, as shown in Figure 3. The test results of three test specimens are shown in Figure 4. The friction coefficient obtained by the fitting curve was 0.58.

### 2.5. Corroded Push Out Test 

Two series of corroded push out test specimens were also tested for comparison. The test specimens were corroded and tested using the same procedure described by Chen [6]. The materials used in the push out test specimens were the same as those used in the single stud test specimens (different from the test specimens used by Chen [6]). The measured corrosion rates of the studs and ultimate strengths are shown in Table 7 and Table 8. The test specimens were labeled so that the nominal stud diameter and expected corrosion rate could be identified from the label. The first letter indicates the nominal diameter of the stud, where the prefix letter “D” refers to the diameter.

## 3. Test Results

### 3.1. Measurement of the Stud Corrosion Rate

**Figure 5:**
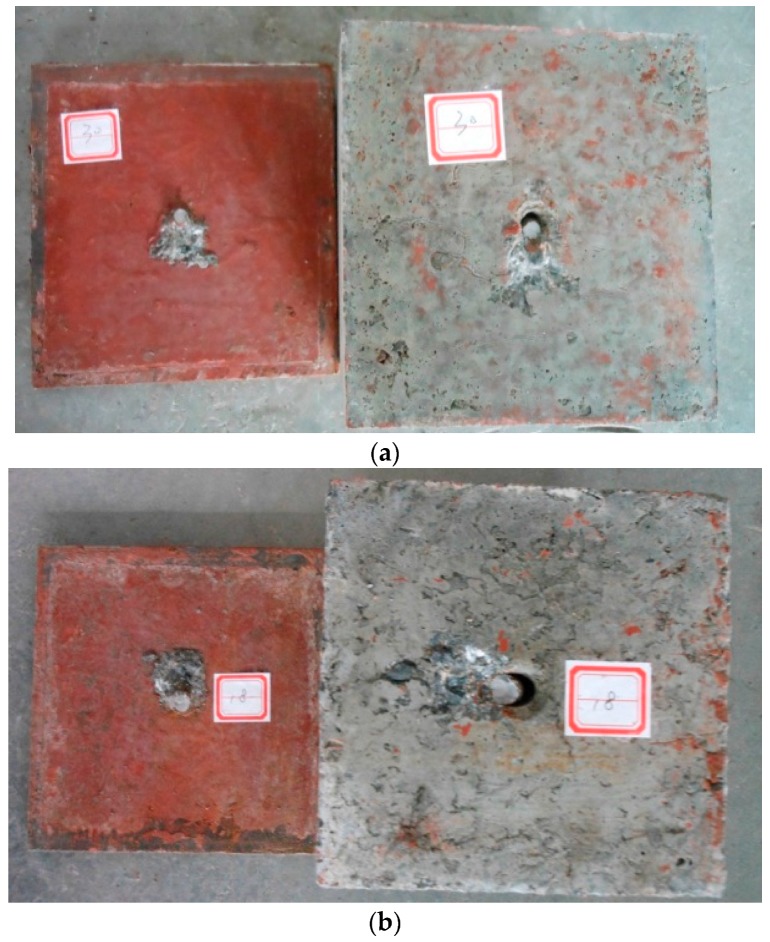
Typical failure mode of the single stud shear connector specimen: (**a**) Specimen W10.0-5; (**b**) Specimen W13.0-10.

**Figure 6:**
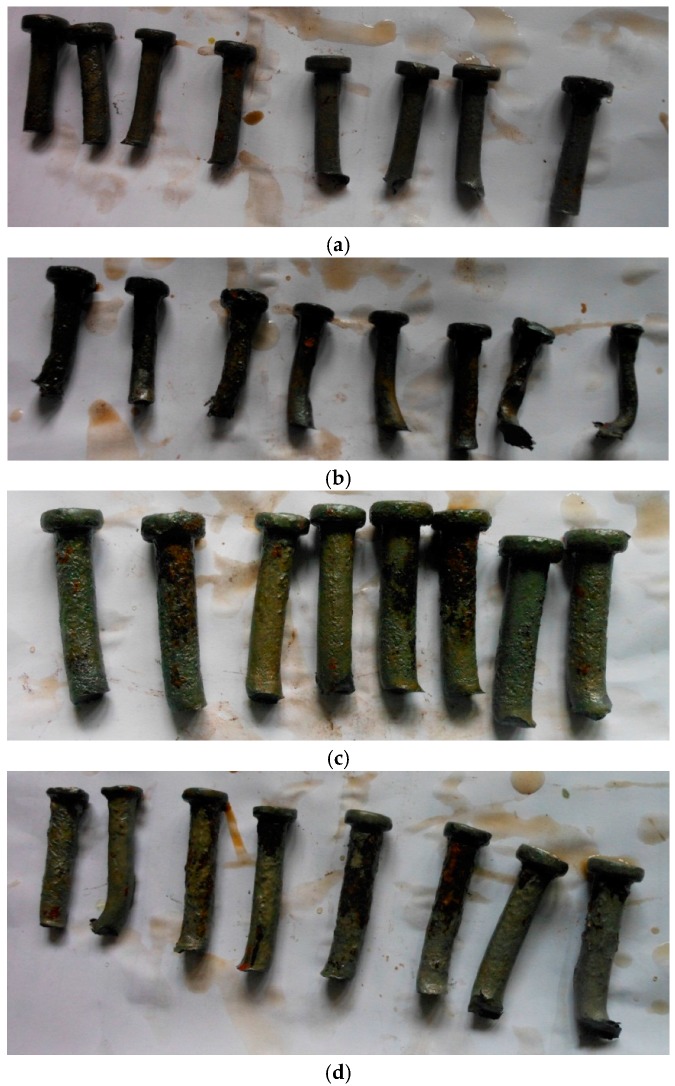
Corroded stud shear connectors: (**a**) 10–30; (**b**) 10–50; (**c**) 13–30; (**d**) 13–50.

The corroded studs were retrieved from the failed specimens (shown in Figure 5) and the corrosion product was cleaned using a corrosion-inhibited HCl solution [24]. The corroded studs having different corrosion rates are shown in Figure 6. The area loss of the steel rebar (∆A) was estimated afterwards by subtracting the post-corrosion area from the measured pre-corrosion area. The post-corrosion area of the stud was calculated using the measured diameter of the shank of the stud. The measured diameter of the shank was used to calculate the corrosion rate of each stud (ψ) as: ψ = (A − ∆A)/A%. For the push out test specimens, the average corrosion rate of eight studs was taken as the corrosion rate of each push test specimen. It is shown that the measured corrosion rates of both the single stud test specimen and the push test specimens are different from the expected corrosion rates. There is no corrosion occurring between the interface of the concrete slab and the steel plate. 

### 3.2. Static Behavior 

The static behavior of the stud connectors can be described using the load–slip curves and ultimate strength. In this study, the effect of corrosion on the static behavior of stud was investigated. 

#### 3.2.1. Load-Slip Curves

**Figure 7:**
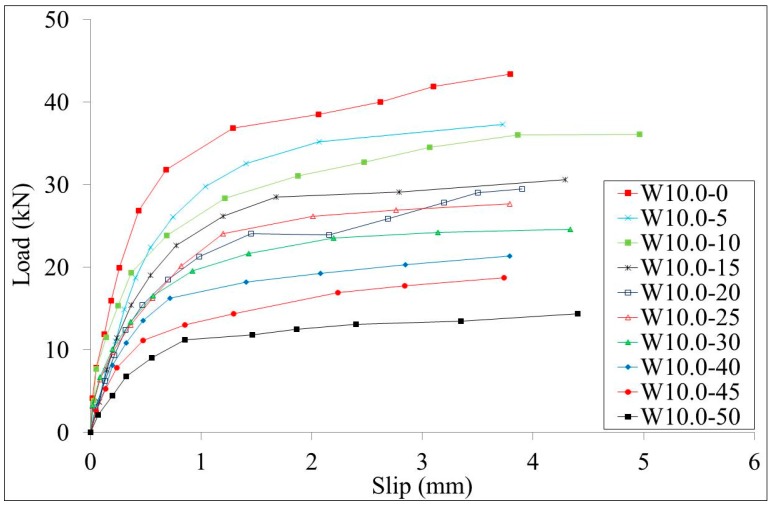
Load-slip curves of the W10.0 series specimens.

**Figure 8:**
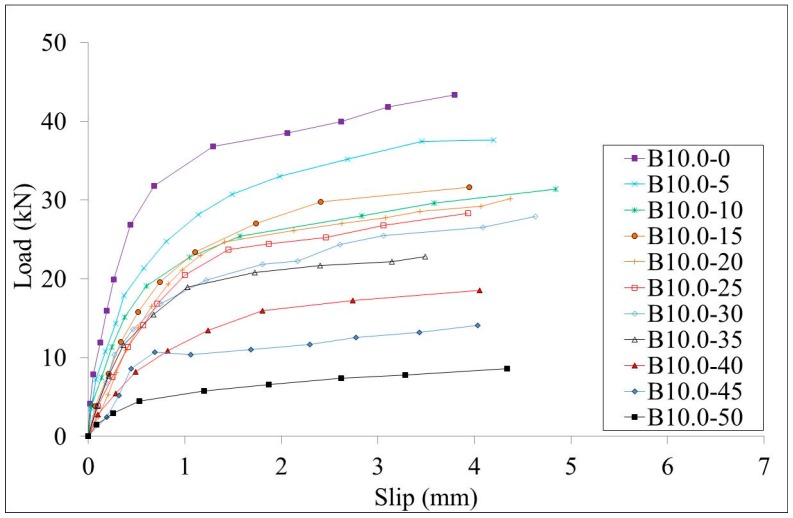
Load-slip curves of the B10.0 series specimens.

**Figure 9:**
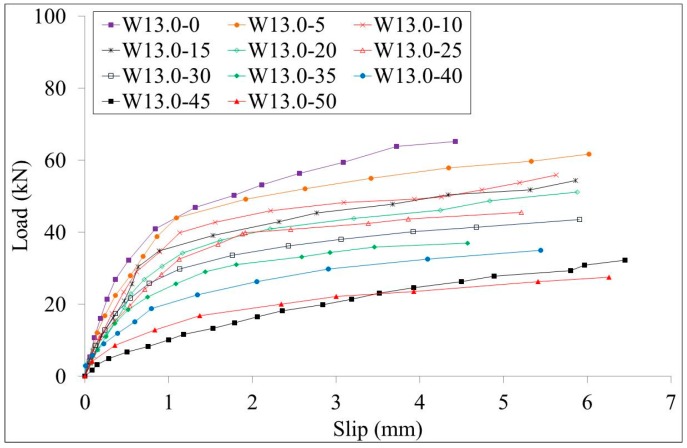
Load-slip curves of the W13.0 series specimens.

**Figure 10:**
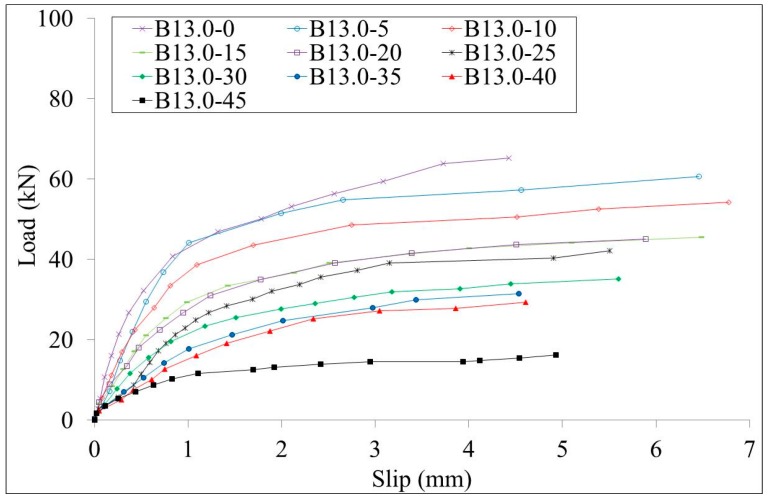
Load-slip curves of the B13.0 series specimens.

The load-slips curves of test specimens W10.0 series and B10.0 series are shown in Figure 7 and Figure 8, respectively. The load-slips curves of test specimens W13.0 series and B13.0 series are shown in Figure 9 and Figure 10, respectively. Since the failure mode of all specimens was the stud failure, the load-slip curves could only be measured up to the point of the ultimate strength. Studs that had corrosion along the whole length and studs that had bottom corrosion showed similar load-slip curves. It is shown that the initial stiffness of the specimens decreases with the increment of the corrosion rate for both series of specimens. The ductility of the specimens showed no obvious relation with the corrosion rates. 

#### 3.2.2. Ultimate Strength

In this study, the failure mode of all push test specimens was the stud failure. Figure 4 shows the typical stud failure of the test specimens. The ultimate strengths of the 10.0 mm diameter test specimen series and the 13.0 mm diameter series are shown in Table 5 and Table 6, respectively. It is shown that the ultimate strengths of the test specimens decrease when the corrosion rate increases. This means that the corrosion has a significant effect on the ultimate strengths of the test specimens.

## 4. Comparison

**Figure 11:**
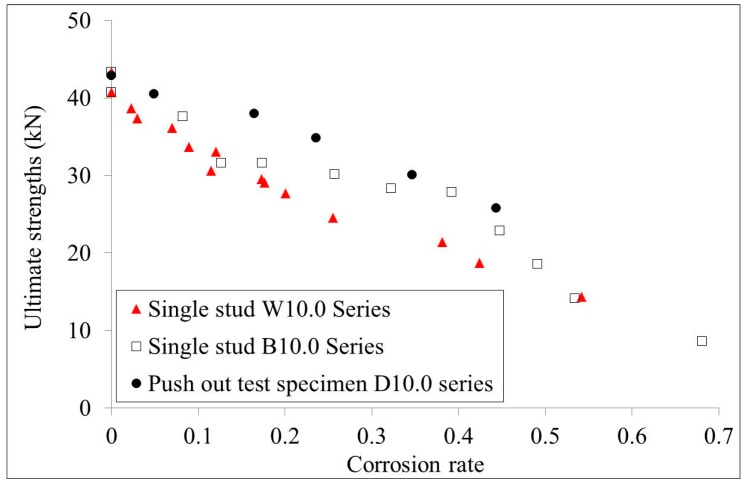
Comparison of the ultimate strengths of the W10.0, B10.0, and D10.0 series specimens.

**Figure 12:**
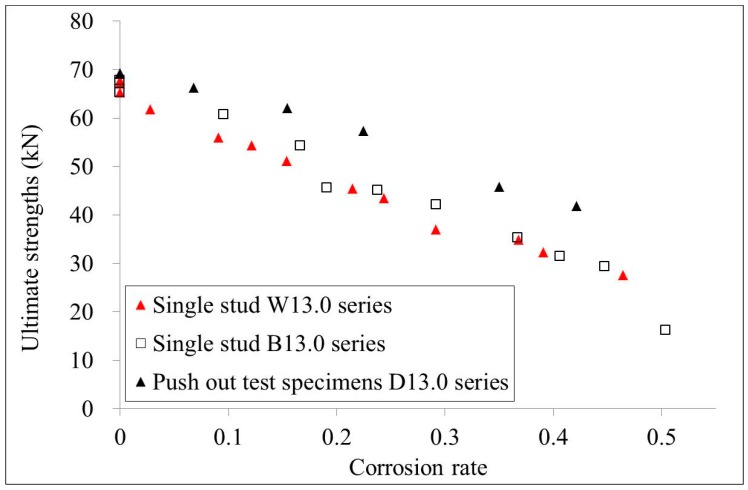
Comparison of the ultimate strengths of W13.0, B13.0, and D13.0 series specimens.

The ultimate strengths of the 10.0 mm diameter single stud test specimen series and the 13.0 mm diameter series were compared with the test results of the push out test specimens, as shown in Figure 11 and Figure 12, respectively. It is shown that the ultimate strengths of the push out test specimens are relatively higher than those of the single stud test specimens that have the same corrosion rate. For specimens with 10.0 mm diameters, studs that had corrosion along the whole length showed lower ultimate strengths compared with those studs that had bottom corrosion. However, for specimens with 13.0 mm diameters, studs that had corrosion along the whole length showed similar ultimate strengths as those studs that had bottom corrosion. Generally, the ultimate strengths obtained from the corroded single stud test specimens are conservative compared with those obtained from the corroded push out test specimens. 

## 5. Conclusions

Experimental investigations of steel and concrete composite single stud shear connector specimens with corrosion deterioration were conducted in this study. Two series of test specimens that had different stud diameters were tested. The test specimens were first corroded by the electronic accelerating method were then loaded to failure. Based on the test results, the effect of corrosion on the load-slip curves and ultimate strength were studied. It was shown that the corrosion of the stud had a significant effect on the ultimate strengths of the test specimens. The test results obtained from the single stud shear connector tests were compared with the test results obtained from the corroded push out test specimens. It was shown that the single stud shear connector tests provided conservative test results. 

## Figures and Tables

**Table 1:** Material properties of concrete.

Specimen	*E_c_*(MPa)	*f_cu_* (MPa)
1	3.32 × 10^4^	45.4
2	3.38 × 10^4^	45.8
3	3.40 × 10^4^	46.7
Average	3.37 × 10^4^	46.0

**Table 2:** Material properties of the stud material.

Specimen	Elastic Modulus (MPa)	Yield Stress (MPa)	Tensile Strength (MPa)	Elongation (%)
10.0 mm	1.94 × 10^5^	462.7	512.0	26.4
13.0 mm	1.98 × 10^5^	431.2	490.6	24.9

**Table 3:** Expected stud corrosion rate and actual corrosion time of the 10.0 mm series.

Specimen	Expected Corrosion Rate (%)	Corrosion Time (Hours)	Measured Corrosion Rate (%)
W10.0-0	0	0	0
B10.0-0			0
W10.0-5	5	461	2.97
B10.0-5			8.23
W10.0-10	10	923	8.93
B10.0-10			12.68
W10.0-15	15	1384	12.01
B10.0-15			17.38
W10.0-20	20	1845	17.65
B10.0-20			25.71
W10.0-25	25	2307	20.06
B10.0-25			32.23
W10.0-30	30	2768	25.55
B10.0-30			39.19
W10.0-35	35	3230	---
B10.0-35			44.78
W10.0-40	40	3691	38.15
B10.0-40			49.09
W10.0-45	45	4152	42.41
B10.0-45			53.43
W10.0-50	50	4614	54.14
B10.0-50			68.09

**Table 4:** Expected stud corrosion rate and actual corrosion time of the 13.0 mm series.

Specimen	Expected Corrosion Rate (%)	Corrosion Time (Days)	Measured Corrosion Rate (%)
W13.0-0	0	0	0
B13.0-0			0
W13.0-5	5	599	2.77
B13.0-5			9.56
W13.0-10	10	1199	9.09
B13.0-10			16.67
W13.0-15	15	1798	12.15
B13.0-15			19.08
W13.0-20	20	2398	15.35
B13.0-20			23.81
W13.0-25	25	2997	21.46
B13.0-25			29.22
W13.0-30	30	3596	24.35
B13.0-30			36.74
W13.0-35	35	4196	29.13
B13.0-35			40.62
W13.0-40	40	4795	36.78
B13.0-40			44.78
W13.0-45	45	5394	39.07
B13.0-45			50.04
W13.0-50	50	5994	46.44
B13.0-50			----

**Table 5:** Ultimate strengths of the 10.0 mm series single stud specimens.

Specimen	Measured Corrosion Rate (%)	Ultimate Strength (kN)
W10.0-0	0	43.37
B10.0-0	0	43.37
W10.0-5	2.97	38.67
B10.0-5	8.23	37.6
W10.0-10	8.93	36.11
B10.0-10	12.68	31.62
W10.0-15	12.01	30.62
B10.0-15	17.38	31.62
W10.0-20	17.65	29.53
B10.0-20	25.71	30.16
W10.0-25	20.06	27.68
B10.0-25	32.23	28.34
W10.0-30	25.55	24.59
B10.0-30	39.19	27.89
W10.0-35	---	---
B10.0-35	44.78	22.86
W10.0-40	38.15	21.38
B10.0-40	49.09	18.54
W10.0-45	42.41	18.75
B10.0-45	53.43	14.09
W10.0-50	54.14	14.38
B10.0-50	68.09	8.61

**Table 6:** Ultimate strengths of the 13.0 mm series single stud specimens.

Specimen	Measured Corrosion Rate (%)	Ultimate Strength (kN)
W13.0-0	0	65.28
B13.0-0	0	65.28
W13.0-5	2.77	61.76
B13.0-5	9.56	60.68
W13.0-10	9.09	55.95
B13.0-10	16.67	54.2
W13.0-15	12.15	54.42
B13.0-15	19.08	45.51
W13.0-20	15.35	51.16
B13.0-20	23.81	45.1
W13.0-25	21.46	45.5
B13.0-25	29.22	42.14
W13.0-30	24.35	43.5
B13.0-30	36.74	35.25
W13.0-35	29.13	37.01
B13.0-35	40.62	31.44
W13.0-40	36.78	34.95
B13.0-40	44.78	29.35
W13.0-45	39.07	32.34
B13.0-45	50.04	16.21
W13.0-50	46.44	27.6
B13.0-50	----	65.28

**Table 7:** Ultimate strengths for the D10.0 series push out test specimens.

Specimen	Measured Corrosion Rate (%)	Ultimate Strength (kN) Test (*P_test_*)
D10.0-0A	0	42.9
D10.0-10	4.93	40.5
D10.0-20	16.44	38
D10.0-30	23.61	34.8
D10.0-40	34.66	30.1
D10.0-50	44.33	25.8

**Table 8:** Ultimate strengths for the D13.0 series push out test specimens.

Specimen	Measured Corrosion Rate (%)	Ultimate Strength (kN) Test (*P_test_*)
D13.0-0A	0	69.3
D13.0-10	6.78	66.3
D13.0-20	15.41	62.1
D13.0-30	22.43	57.3
D13.0-40	34.99	45.8
D13.0-50	42.12	41.9

## Nomenclature

*A*	=	cross sectional area of the stud;
Δ*A*	=	cross sectional area loss of the stud;
*d*	=	the diameter of the shank of the stud;
*d_c_*	=	the diameter of the shank of the corroded stud;
*E_c_*	=	the elastic modulus of the concrete at 28 days;
*E_cm_*	=	the elastic modulus of the concrete slab;
*f_ck_*	=	the characteristic cylinder compressive strength of the concrete at the age considered;
*f_cu_*	=	the compressive strength of the concrete at 28 days;
*f_u_*	=	specified ultimate tensile strength of the material of the stud;
*P_test_*	=	ultimate strength obtained from the test results;
ψ	=	corrosion rate of the stud.
